# Emergency Department Management of Ankle Fractures: A Narrative Review

**DOI:** 10.7759/cureus.116211

**Published:** 2026-09-14

**Authors:** Micah Ngatuvai, Francis Villarreal, Victor M Vasquez, Nate Dammann, Mohammed Pourghaed, Brandon Chung

**Affiliations:** 1 Orthopaedic Surgery, William Beaumont Army Medical Center, Fort Bliss, USA; 2 Orthopaedic Surgery, Texas Tech University Health Sciences Center El Paso Paul L. Foster School of Medicine, El Paso, USA; 3 Orthopaedic Surgery, Liberty University College of Osteopathic Medicine, Lynchburg, USA

**Keywords:** ankle fractures, ankle injury, danis-weber classification, emergency department, fracture reduction, immobilization, lauge-hansen classification, orthopedic trauma, pain management

## Abstract

Ankle fractures are among the most common orthopedic injuries encountered in the emergency department. Nearly half occur secondary to low-energy mechanisms such as ground-level falls, and approximately one-fourth ultimately require surgery. Appropriate acute management and disposition by providers in the acute setting is crucial to optimizing patient outcomes. This begins with a general knowledge of the commonly used Danis-Weber and Lauge-Hansen classification system, which helps categorize the majority of ankle fracture patterns encountered. Initial management includes timely reduction, multimodal pain control, and appropriate immobilization. Available resources, provider experience, and interfacility practice patterns influence specific management decisions; however, multiple effective treatment strategies exist. This review provides a concise resource regarding emergency department management and the different reduction maneuvers that can be utilized.

## Introduction and background

Ankle fractures are among the most common fractures presenting to U.S. hospitals, with approximately 673,000 cases treated in emergency departments over a five-year period, corresponding to an incidence of 4.22 per 10,000 person-years [1]. Surgical treatment adds substantial healthcare costs, with average commercial payments for closed ankle fracture fixation estimated at USD $16,775 per case and inpatient surgeries costing USD $28,169 per injury [2]. Proper initial management is crucial for optimizing patient outcomes and preventing complications. Understanding ankle fracture classification systems is essential for guiding appropriate treatment. The Danis-Weber classification categorizes fractures based on the level of fibular injury relative to the syndesmosis, the ligamentous complex connecting the distal tibia and fibula, while the Lauge-Hansen system classifies fractures according to foot position and deforming force at the time of injury [3,4]. Although a comprehensive discussion of classification systems is beyond the scope of this review, familiarity with these systems and the ankle mortise, the osseous articulation formed by the distal tibia and fibula surrounding the talus, is fundamental to patient management. This review focuses on the two main classification systems used to guide ankle fracture treatment and evidence-based approaches to pain control, reduction techniques, and immobilization strategies in the emergency department setting. Furthermore, this study seeks to highlight commonly used ankle reduction maneuvers in a single review, providing a critical resource to aid in the management of acute ankle fractures.

## Review

Methodology

A literature search was conducted regarding emergency department management of ankle fractures. A comprehensive search of PubMed, MEDLINE, and Google Scholar databases was performed for studies published through March 2026. Search terms included combinations of “ankle fracture,” “ankle fracture reduction,” “ankle dislocation,” “emergency department ankle management,” “splinting,” “ankle block,” “hematoma block,” “Lauge-Hansen,” “Danis-Weber,” and “ankle fracture immobilization.”

Articles were included if they discussed acute ankle fracture classification, reduction techniques, and immobilization strategies. Review articles, cadaveric studies, clinical trials, technical notes, and education resources were considered. Articles not available in English, studies unrelated to acute ankle fracture management, and publications focused solely on postoperative rehabilitation or chronic ankle instability were excluded.

Relevant studies were screened by title and abstract for applicability to the topic. Full-text review was subsequently performed for selected articles. Data were synthesized narratively with emphasis placed on clinically applicable reduction techniques, emergency management principles, and immobilization strategies. Given the heterogeneity of the included literature and the educational focus of this manuscript, a formal risk-of-bias assessment and meta-analysis were not performed.

Initial assessment and evaluation

The Ottawa Ankle Rules are widely used in emergency care to guide decisions about radiographic evaluation and to help minimize unnecessary imaging in patients with acute ankle injuries [3]. Specifically, ankle radiographs are recommended when there is bony tenderness along the posterior edge or tip of either malleolus, or when the patient is unable to bear weight both immediately after the injury and during assessment in the emergency department [3,4]. Initial evaluation of an ankle fracture should prioritize assessment of the overlying skin and soft tissues, as the presence of open wounds, tenting, or significant swelling may necessitate urgent intervention and directly influence the timing of definitive fixation or the decision to temporize with an external fixator. Severe soft-tissue compromise, open injuries, or threatened skin integrity may warrant urgent orthopedic consultation and, in select cases, involvement of plastic surgery services [5]. A thorough neurovascular examination is equally essential, with careful documentation of motor and sensory function and distal perfusion. Radiographic evaluation should include anteroposterior (AP), mortise, and lateral views to define the fracture pattern, assess stability, and guide reduction [5]. Additional views of the tibia/fibula and foot are often warranted to exclude concomitant injuries as per standard trauma series radiographs. Of note, advanced imaging and orthopedic consultation may be indicated for complex fracture patterns, and definitive management of unstable or substantially displaced injuries often involves operative fixation [5].

Fractures are broadly classified as stable or unstable based on radiographic criteria. Stable fractures demonstrate no medial clear space widening, no talar subluxation, and appropriate tibiofibular overlap and clear space; these may be managed with a CAM boot and outpatient follow-up [5]. Unstable fractures, characterized by medial clear space widening, talar subluxation, or disruption of the mortise joint or syndesmosis, require closed reduction and splinting before definitive management. In cases of irreducible fracture patterns, significant soft-tissue disruption, or open injuries, a temporizing external fixator is warranted before discharge [5].

The goal of reduction is to restore anatomic alignment, relieve soft-tissue pressure, and preserve long-term joint function. This requires relocating the talus beneath the tibial plafond, restoring fibular length and alignment, and correcting articular incongruity. Even minor displacement carries significant consequences: 1 mm of lateral talar shift reduces tibiotalar contact area by 42%, substantially increasing joint contact pressures and the risk of post-traumatic arthritis [6,7]. Temporary stability is maintained with splinting, while definitive management is guided by fracture stability, degree of displacement, soft-tissue status, and patient comorbidities [5].

Clinical classification of ankle fractures

Classification systems provide a standardized framework for communication, documentation, and research comparison. They provide a standardized language that facilitates clear communication among providers, ensures consistency in documentation, and enables meaningful comparison of outcomes across research populations. Additionally, well-validated classification systems carry diagnostic value by helping clinicians anticipate associated ligamentous and osseous injuries not apparent on initial imaging. Below, we review the two most commonly used classification systems used for ankle fractures.

Danis-Weber Classification

The Danis-Weber classification organizes a fibula fracture based on the fracture location in relation to the syndesmosis [8]. Type A fractures occur below the syndesmosis, type B at the level of the syndesmosis, and type C above the syndesmosis (Figure 1).

**Figure 1:**
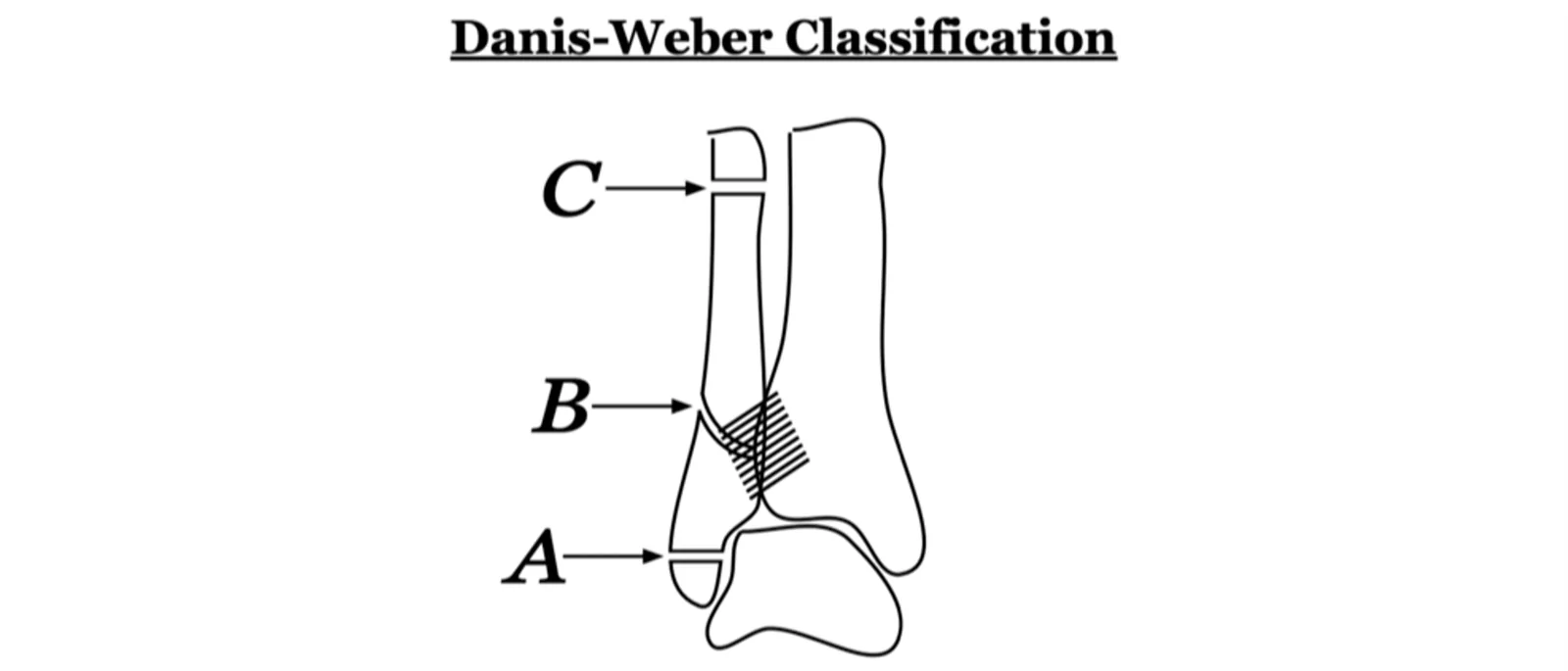
Illustration representing the Danis-Weber classification. Line A represents a fracture below the syndesmosis, line B represents a fracture at the level of the syndesmosis, and line C represents a fracture above the syndesmosis. Authors’ own image. No artificial intelligence tools were used in preparing the figure.

Associated syndesmotic injury increases in frequency from type A to type C fractures, with syndesmosis disruption present in <1% of type A, 52% of type B, and 92% of type C fractures [9]. Isolated fibula fractures, particularly Danis-Weber type B and C, often require a stress examination to assess for mortise disruption when it is not clear on the initial X-rays. Stress examination options include manual stress, gravity stress, and/or weight-bearing radiograph [8].

Lauge-Hansen Classification

The Lauge-Hansen classification describes ankle fractures based on foot position and direction of force at the time of injury [8]. There are four injury patterns, namely, supination-external rotation (SER) (most common), pronation-external rotation (PER), supination-adduction (SAD), and pronation-abduction (PAB) (Figure 2).

**Figure 2:**
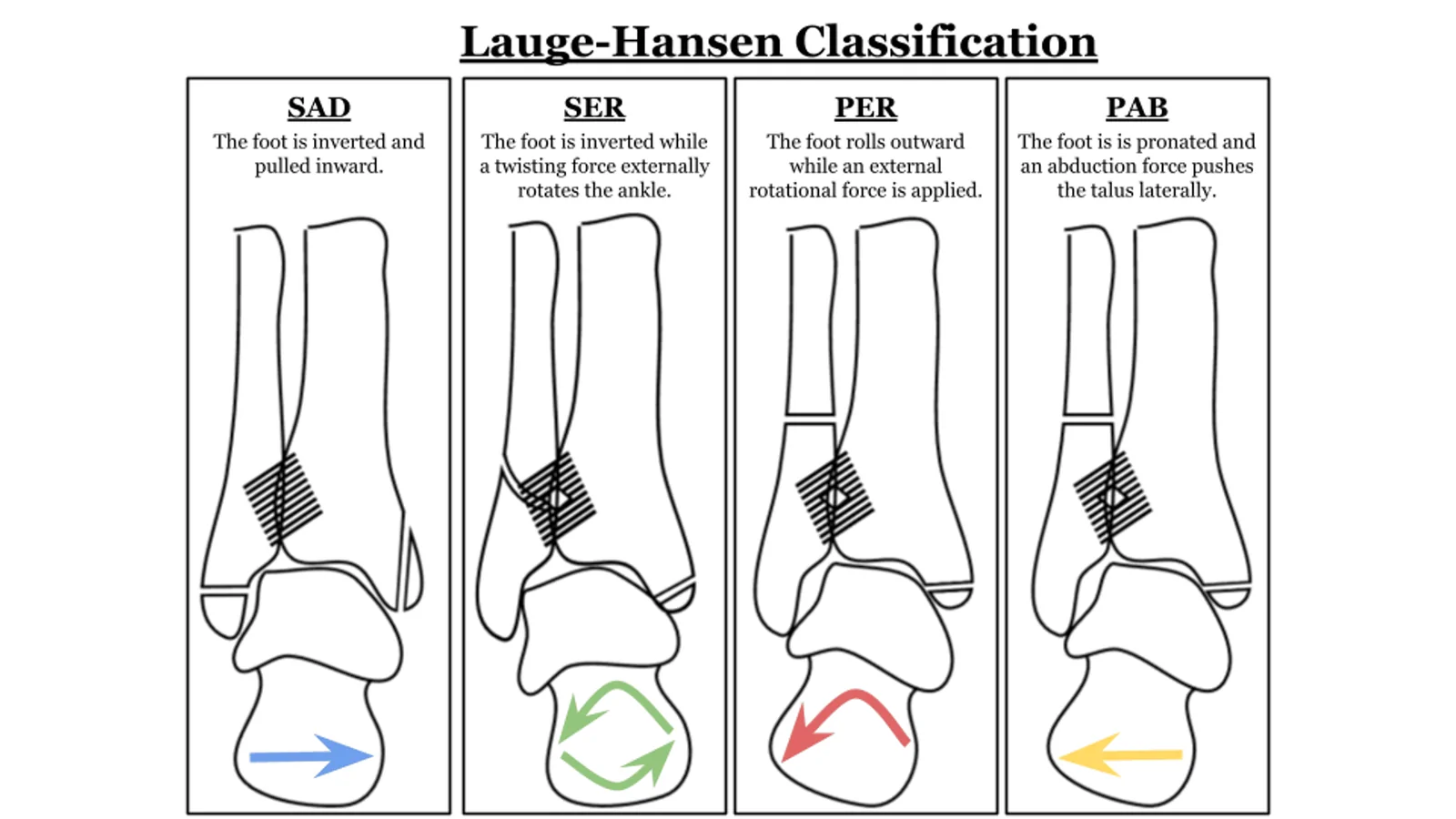
Visual depiction regarding the Lauge-Hansen classification and different mechanisms of injury. Authors’ own image. No artificial intelligence tools were used in preparing the figure.

Lauge-Hansen predicts expected osseous and ligamentous injury in a stepwise pattern based on compression and tension forces during the time of injury [8]. All Lauge-Hansen pattern types except SAD most commonly lead to lateral talar shift and medial clear space widening, requiring reduction with lateral (medial-directed) force at the talus. SAD fracture patterns lead to a vertical medial malleolus fracture, leading to disruption of the medial buttress and an uncommon medial talar shaft, requiring mold reversal with medial (lateral-directed) force at the talus [8].

Acute pain management

Regional anesthesia is central to early pain control in patients presenting with ankle fractures [10]. The ankle block is the most commonly used technique, providing anesthesia to the deep peroneal, tibial, superficial peroneal, sural, and saphenous nerves for fracture reduction, wound care, and perioperative management [10]. Short-acting agents such as lidocaine offer rapid onset within approximately 5-10 minutes and 20-30 minutes of anesthesia, extendable to two hours with epinephrine; the maximum adult dose is 300 mg, or 500 mg with epinephrine [11]. For prolonged analgesia, bupivacaine 0.25-0.5% provides four to six hours of anesthesia (up to eight hours with epinephrine), with maximum doses of 175 mg or 225 mg when combined with a vasoconstrictor. Lidocaine-bupivacaine mixtures are commonly used to achieve both rapid onset and extended duration during urgent reductions. Ultrasound guidance may improve accuracy, though landmark-based techniques remain commonly utilized in acute trauma settings [12,13].

Surface anatomy allows efficient localization of these nerves. The deep peroneal nerve is accessed between the extensor hallucis longus and extensor digitorum longus tendons near the ankle joint, typically just lateral to the anterior tibial artery in more than 50% of patients [13]. The tibial nerve lies posterior to the posterior tibial artery at the medial malleolus at a depth of 8-10 mm. The superficial peroneal, sural, and saphenous nerves are anesthetized through subcutaneous infiltration along their predictable courses around the malleoli [10,13]. When rapid dorsal foot anesthesia is required, the common peroneal nerve can be targeted at the fibular neck, often confirmed by a Tinel’s sign radiating to the lateral leg and dorsum of the foot [14].

Despite the advantages of regional anesthesia, the hematoma block remains a reliable and widely used technique for analgesia during ankle fracture reduction [15,16]. With the ankle slightly plantarflexed, the anterior joint line is accessed through the anteromedial portal between the tibialis anterior tendon and medial malleolus. After aspiration confirms hematoma entry, 5-12 mL of 0.5-1% lidocaine without epinephrine is injected. Bupivacaine may be used when prolonged anesthesia is required, though caution is warranted due to its higher cardiotoxicity and chondrotoxicity [15,17,18].

When regional or local anesthesia is inadequate or contraindicated, systemic pharmacologic options are employed. Short-acting opioids such as fentanyl (0.5-1 µg/kg every two to three minutes) and morphine (2.5-10 mg every one to four hours) provide rapid analgesia. Conscious sedation, reserved for opioid-intolerant or hyperalgesic patients, may be achieved with propofol (0.5-1 mg/kg with 0.25-0.5 mg/kg boluses) or midazolam (0.5-2.5 mg every two to five minutes), often in combination with narcotics as appropriate [19-21].

Reduction techniques and positioning

Goals of Reduction

Reduction is most effectively performed with the knee flexed to relax the gastrocnemius-soleus complex and minimize deforming forces during the maneuver [5,22]. The direction of talar shift largely dictates the appropriate reduction position and molding strategy [6,22]. While many ankle fractures can be reduced by proper positioning, some may require additional molds to be placed after splint placement. In the most common scenario, lateral talar shift, one hand is placed at the mid-tibia applying a medially directed force along the medial aspect of the leg, while the other hand is positioned on the lateral aspect of the ankle and calcaneus, providing a lateral-to-medial corrective force. This corrective force is maintained until the splint hardens, avoiding excessive pressure or wrinkles that can cause skin breakdown. This two-handed technique effectively translates the talus beneath the tibial plafond and is the most commonly used maneuver in clinical practice [5,22]. In cases where the talar shift is directed medially, such as in an SAD pattern ankle fracture, the direction of applied forces is reversed accordingly [8].

Different fracture patterns demand additional specific considerations. SER injuries may be amenable to gravity-assisted reduction [23], while bimalleolar and trimalleolar injuries frequently require meticulous restoration of fibular length and precise realignment of the talus [7,24]. Fracture-dislocations, in particular, mandate prompt reduction to alleviate tension on the overlying skin and reduce the risk of neurovascular compromise. Failure to achieve adequate reduction predisposes patients to chronic instability, residual deformity, and early-onset post-traumatic arthritis [24]. The choice of reduction technique should therefore be guided by the fracture pattern, available staffing, patient body habitus, the presence of additional injuries, and the need for sedation. Providers should explicitly weigh these factors before any reduction attempt, as no single technique is universally optimal [25].

Assistant-Aided Reduction

The assistant-aided reduction technique involves an assistant flexing the patient’s hip and knee to approximately 90 degrees, thereby relaxing the gastrocnemius-soleus muscle complex and reducing its deforming forces on the ankle joint (Figure 3).

**Figure 3:**
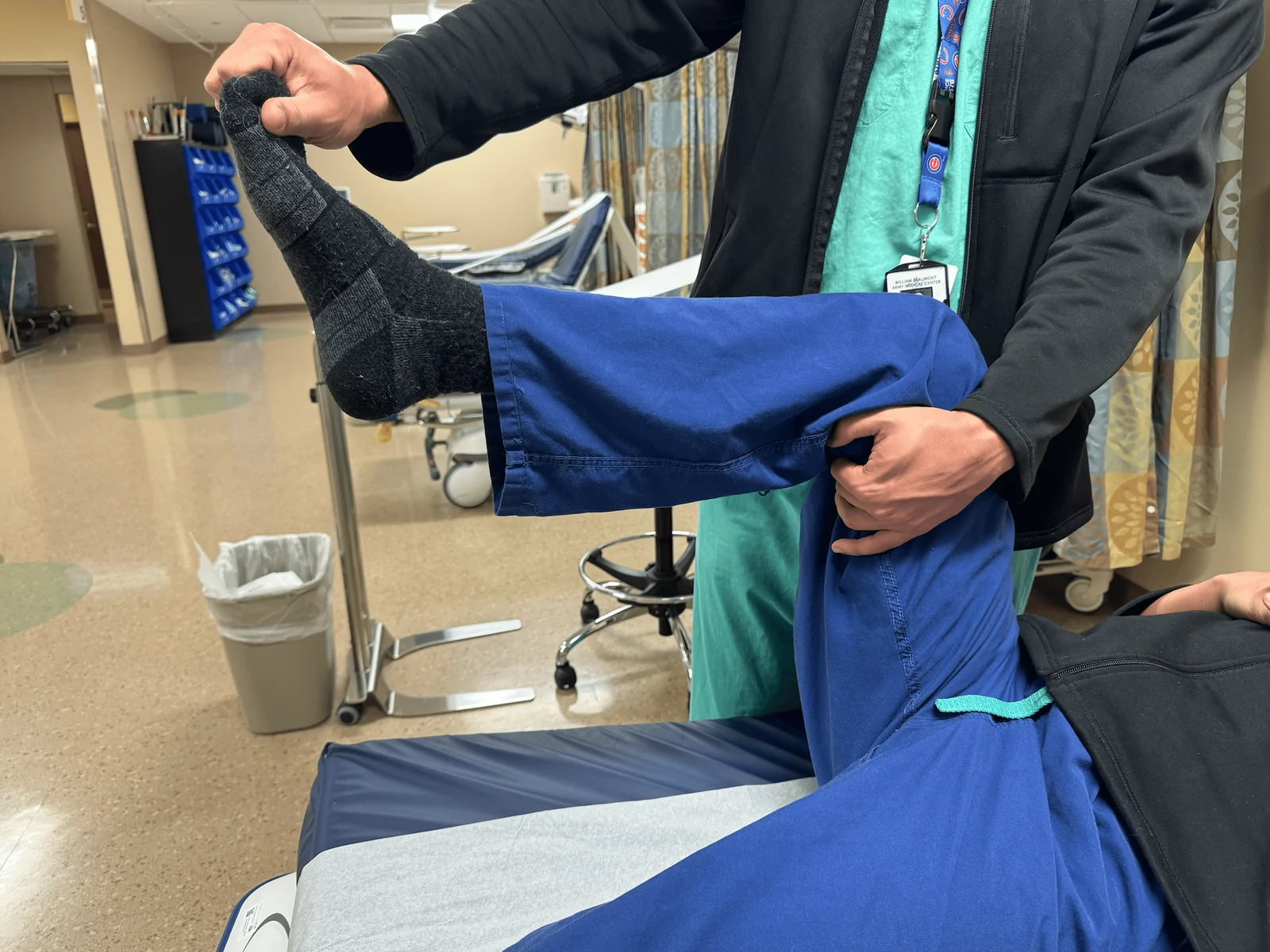
Assistant-aided ankle reduction with the hip and knee flexed to 90 degrees. An assistant flexes the patient’s hip and knee to approximately 90 degrees, which relaxes the gastrocnemius-soleus complex and facilitates improved control during ankle reduction.

This position facilitates mechanical control during manipulation and splint application, proving especially advantageous for unstable fracture patterns requiring manual reduction [25]. The assistant-aided method is particularly suitable for complex fractures where maintaining reduction throughout splinting is challenging [7,25].

Modified Quigley Technique

The modified Quigley technique allows the provider to independently position the leg without requiring additional personnel, accommodating patients across all body types and sizes [26]. With the patient supine, a continuous stockinette is placed under the knee and run distally along the leg, wrapping around the toes to suspend the entire extremity. The proximal end can be managed several ways depending on patient ability and available resources: held by the patient themselves, tied behind their back, or secured to a fixed structure behind them such as an IV pole or overhead light handle (Figure 4).

**Figure 4:**
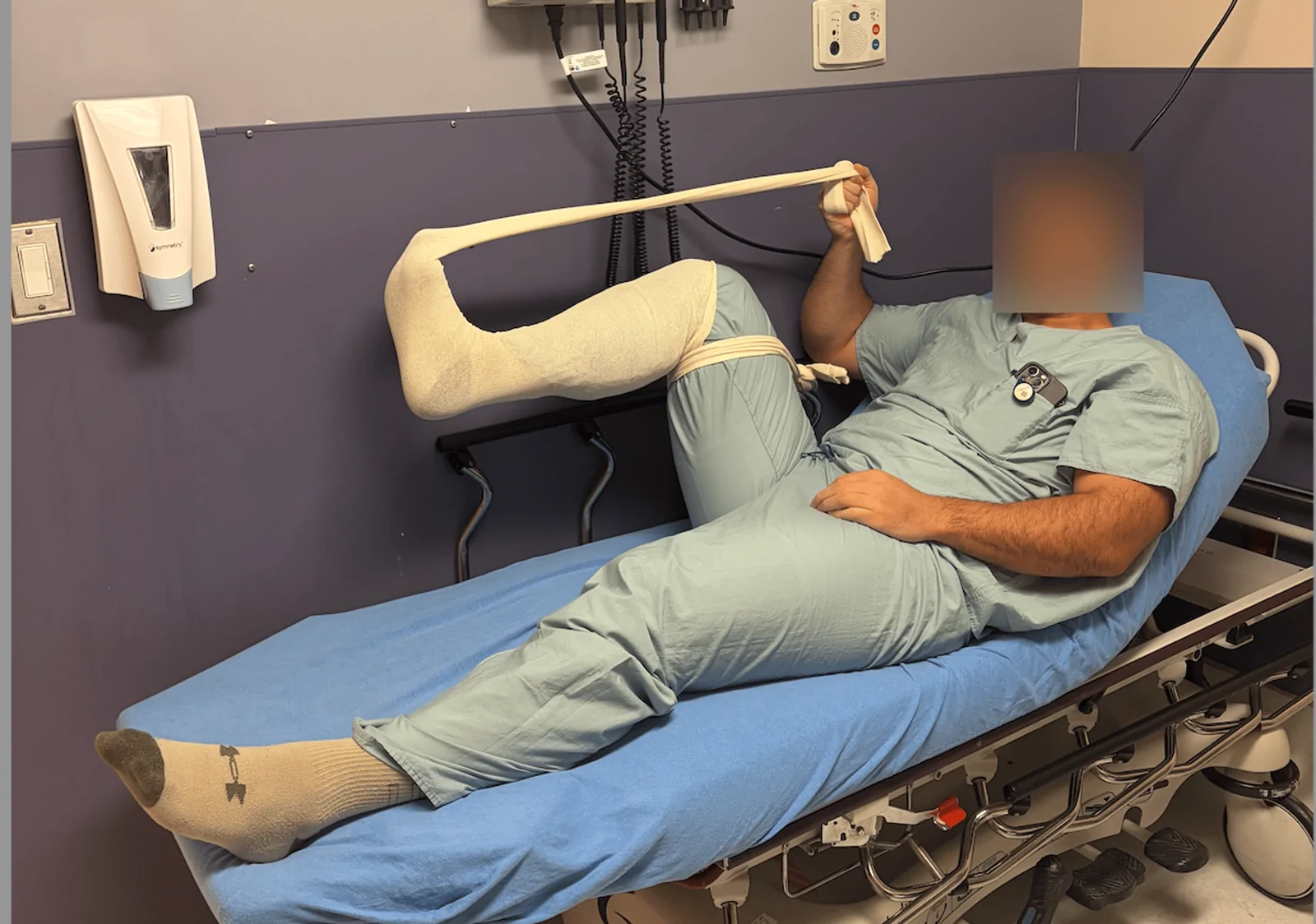
Modified Quigley technique using stockinette suspension. A continuous stockinette is placed under the knee, extended distally around the foot and toes, and used to suspend the extremity. The proximal end is held by the patient to maintain traction, allowing independent positioning of the leg during reduction.

Alternatively, gauze can be secured around the first and second toes, and, in a similar fashion, this can be secured to an IV pole or a similar object. Once suspension is achieved, a separate stockinette is placed under the affected thigh and directed according to the deformity type: for varus deformities, the ends are pulled over the ipsilateral shoulder, whereas for valgus deformities, they are directed over the contralateral shoulder. The thigh stockinette is then secured to a rail or the end of the stretcher with four to six square knots, bringing the knee into 90 degrees of flexion (Figure 5) [25-27].

**Figure 5:**
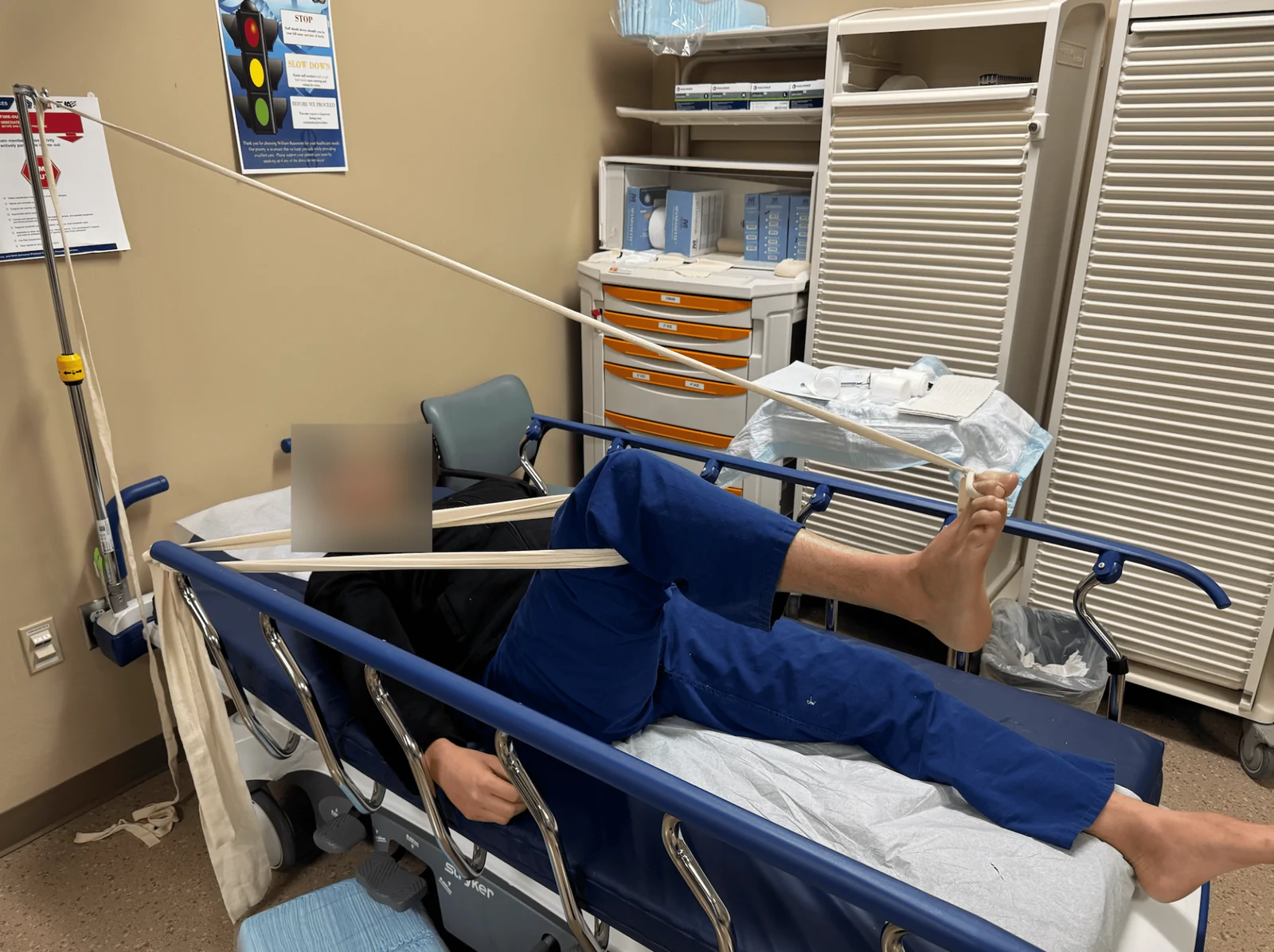
Modified Quigley technique with thigh stockinette positioning and fixation. A second stockinette is placed under the affected thigh and directed according to deformity, then secured to the stretcher to maintain traction. This configuration brings the knee into approximately 90 degrees of flexion and allows controlled positioning of the extremity during reduction.

This sequence, distal suspension before proximal stabilization, ensures the leg remains elevated throughout the procedure and reduces reliance on an assistant. The technique is particularly well suited for SER fractures without gross comminution and is applicable to heavier patients and varus or valgus deformities while minimizing soft-tissue injury. However, highly unstable or open fractures with severe displacement continue to require direct manual reduction and often operative fixation [7,28].

Prone Reduction Technique

Conversely, if the patient can tolerate lying in the prone position, the provider can assist the patient with turning over and bringing the knee to 90 degrees of flexion. This will also allow the ankle to dorsiflex to a neutral position (Figure 6) [27].

**Figure 6:**
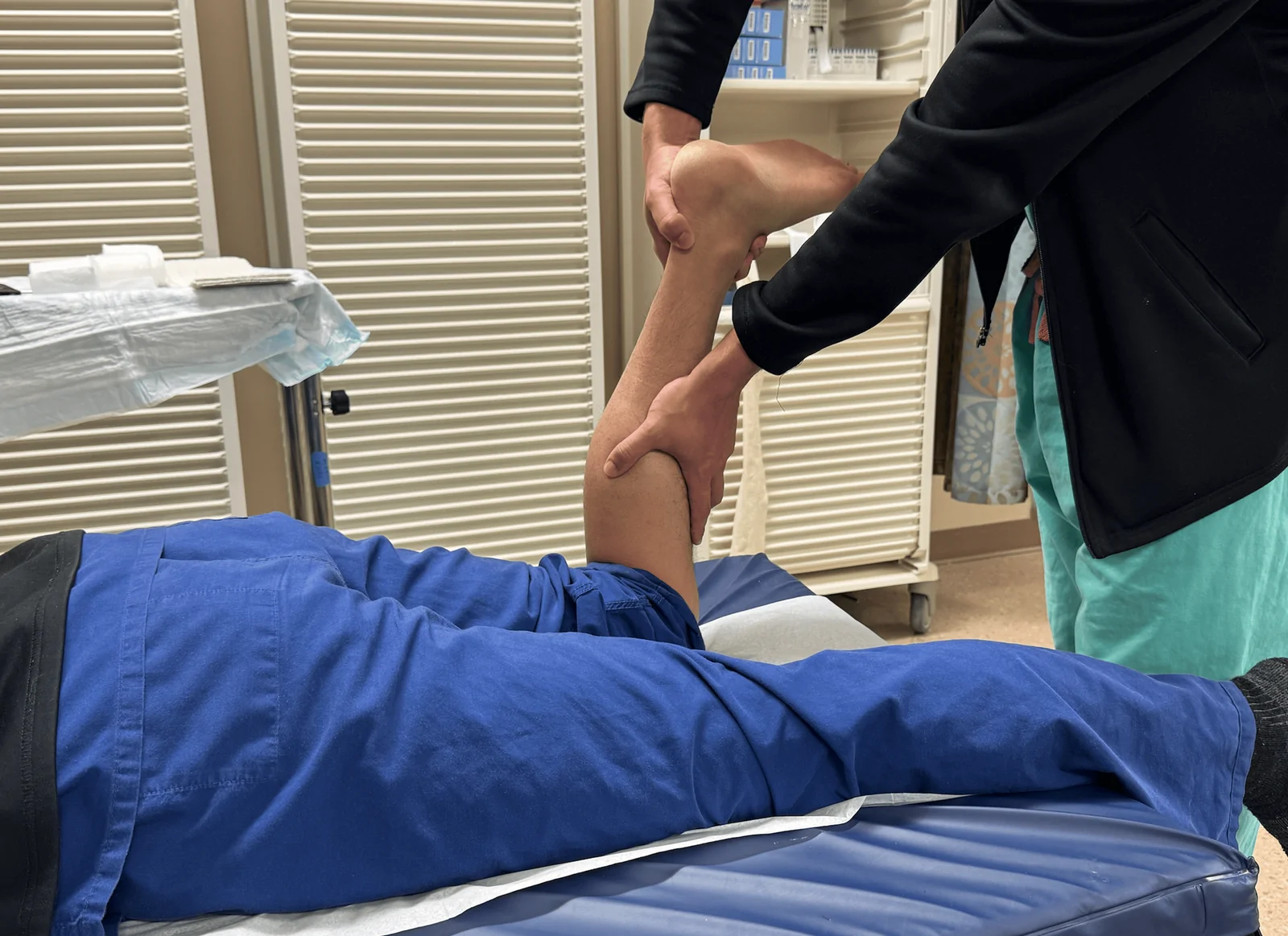
Prone positioning for ankle reduction with the knee flexed to 90 degrees. With the patient in the prone position, the knee is flexed to approximately 90 degrees, allowing the ankle to rest in a neutral dorsiflexed position. This setup facilitates reduction and splinting by using gravity to help maintain alignment.

This position also allows for relative ease of splinting, as the primary splinting material (plaster or fiberglass) will often stay in place due to gravity [27].

Gravity-Assisted Reduction of Ankle

The gravity-assisted reduction of ankle (GARA) technique employs the natural weight of the limb to effect closed reduction, commonly applied to SER ankle fractures [23]. Patients are positioned supine or lateral with the affected leg hanging freely and the knee flexed at 90 degrees (Figure 7).

**Figure 7:**
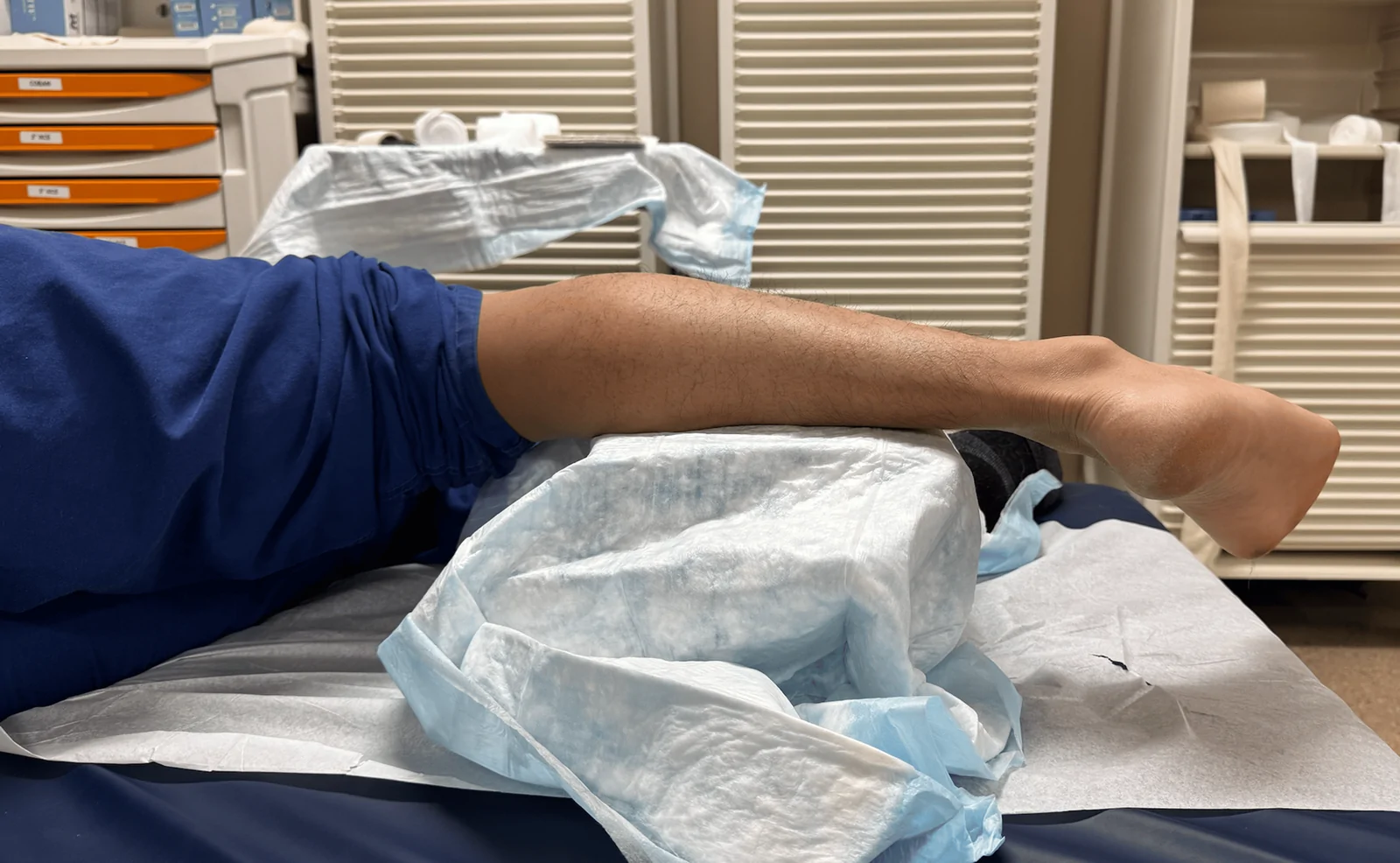
Gravity-assisted reduction of the ankle with dependent positioning. The affected leg is positioned with the ankle hanging freely off the edge of the stretcher while the knee is flexed to approximately 90 degrees. The natural weight of the limb provides gentle traction, facilitating alignment through gravity-assisted reduction.

This low-resource, single-provider method uses gravity to provide gentle traction and facilitate realignment within minutes of positioning [23]. Its noninvasive nature and minimal need for sedation make it ideal for stable or minimally displaced SER fractures, especially in resource-limited settings or in patients intolerant of forceful manipulations. However, GARA is less effective for fractures with gross instability or multi-fragmentary patterns, such as trimalleolar fractures or those with syndesmotic disruption, where manual reduction is often indispensable [7,23].

Immobilization strategies

Splinting Options

The “L&U” splint is commonly utilized by emergency and orthopedic physicians alike as a practical technique for immobilization and stabilization of the acutely reduced ankle. A soft wrap is used with a general guideline of three to four wraps around the distal end, followed by traveling proximally with a 50% overlap in Webril, followed by an additional three to four wraps at the most proximal end [29]. Care is taken to then provide extra padding to the bony prominences such as the heel or medial and lateral malleoli. A posterior slab, “backslab,” or the “L-slab” is applied beginning 2-3 cm distal to the popliteal fossa and extending approximately 1 cm beyond the great toe. This is supplemented by a “U-slab” that encases both malleoli, commencing 2-3 cm distal to the fibular head to avoid iatrogenic injury to the common peroneal nerve, wrapping around the plantar aspect of the foot, and terminating medially at the same level [29,30]. Cotton padding secures both slabs and is loosely reinforced with an elastic bandage to preserve neurovascular status. It is important to note that some providers may also elect to place simply the backslab “L” portion or the “U” portion alone guided by the intended degree of stabilization.

Physician reduction after splint placement is largely dependent on the fracture pattern and patient positioning. Most commonly, the patient is splinted in the supine position, in which the provider may use their chest against the plantar aspect of the foot to control dorsiflexion while using their hand to place the proper valgus or varus stress to the leg [29,30].

Post-reduction

Following immobilization and reduction, repeat radiographic assessment is essential to verify reduction quality. Standard three-view ankle radiographs, i.e., AP, mortise, and lateral, should be obtained to evaluate for residual medial clear space widening, lateral talar shift, anteroposterior translation, and/or syndesmotic widening. Fractures demonstrating adequate reduction should be promptly referred to an orthopedic surgeon for elective outpatient surgical fixation. Cases in which satisfactory reduction cannot be achieved or maintained warrant urgent consultation with the on-call orthopedic surgeon to determine whether repeat closed reduction or temporizing external fixation is appropriate before discharge. Pilon and other intra-articular fracture patterns should be temporized with an external fixator to facilitate ligamentotaxis of the principal fracture fragments [30,31].

Computed tomography (CT) of the ankle should be obtained for all intra-articular and trimalleolar fractures following reduction and immobilization before discharge. This facilitates preoperative planning and ensures appropriate surgical approach and fixation of each fracture fragment. In cases where an irreducible injury pattern necessitates external fixator application, CT should be deferred until after temporization to avoid unnecessary repeat radiation exposure [31].

Complications and prevention

Pressure necrosis can develop in as little as two hours when padding is inadequate, particularly over bony prominences such as the medial malleolus, lateral malleolus, and calcaneus [32]. To reduce the risk, it is recommended to apply at least two to three layers of cast padding, extending 2-3 cm beyond the edges of the splint or cast to prevent pressure points and skin compromise [32]. Patients should also be counseled to keep the heel elevated and to avoid prolonged pressure on the posterior heel, as research has demonstrated that pressure-induced necrosis can occur regardless of the amount of padding used [33].

Compartment syndrome is a rare but serious complication of ankle splinting, especially when immobilization is applied too tightly or swelling progresses after high-energy injuries [34-37]. Bimalleolar, trimalleolar, and pilon (tibial plafond) fractures are most commonly associated with increased compartment syndrome risk due to extensive soft-tissue injury and swelling [35,36]. Serial neurovascular assessments before and after splint application are imperative in these cases.

Thermal burns can occur during plaster or fiberglass curing due to exothermic reactions. Skin burns have been reported at temperatures exceeding 49°C if contact is maintained for more than two to three minutes [38]. Several factors increase this risk, including warm dip water, applying thick layers of plaster or fiberglass, and poorly padded splints. Studies show that increasing the cast padding from two layers to four or more can reduce skin surface temperatures by approximately 8°C, significantly decreasing burn risk. Therefore, clinicians should use cool water (<24°C) for plaster activation, avoid excessive casting thickness, and ensure proper padding placement [38].

Equinus contracture can develop when the ankle is immobilized in plantar flexion for prolonged periods, resulting in shortening of the gastrocnemius-soleus complex and structural changes to the joint capsule and ligaments [39]. This complication can begin to occur after as little as 7-14 days of immobilization, although the risk increases substantially with longer durations [39]. To prevent this, the ankle should be immobilized in neutral dorsiflexion unless there is a specific clinical indication for plantarflexion, such as tendon repairs or acute soft-tissue injuries requiring edema control. Early range of motion and minimizing unnecessary immobilization are key strategies to reduce contracture formation [5,39].

Limitations

This review is not without limitations. Ankle fracture management is a broad and complex topic, and the scope of this review was intentionally limited to provide a concise, clinically accessible overview rather than an exhaustive analysis. As such, certain nuances in fracture classification, reduction technique, and post-reduction management were addressed only superficially. Additionally, the variety of provider-specific tips, positional modifications, and technique variations that exist in practice could not be comprehensively included given the constraints of a focused narrative review. Despite these limitations, we believe this review makes a meaningful contribution to the literature, offering providers a practical visual reference to assist with management of acute ankle fractures.

## Conclusions

Ankle fractures represent a frequent and costly burden on emergency departments, and their initial management has a substantial impact on both immediate complications and long-term outcomes. Classification systems are foundational to this process; the Danis-Weber system provides a practical radiographic framework for assessing stability and syndesmotic involvement, while the Lauge-Hansen classification offers mechanistic insight into associated ligamentous and osseous injuries. Effective pain control, particularly through regional anesthesia techniques such as ankle and hematoma blocks, is a prerequisite for successful reduction and patient cooperation in high-volume settings. Reduction and immobilization strategies should be tailored to fracture stability, degree of displacement, and available personnel. While assistant-aided reduction remains standard for unstable injuries, unassisted and gravity-based methods offer viable alternatives when staffing or sedation resources are limited. Posterior slab and U-splint constructs are widely recommended for acute immobilization given their ability to accommodate swelling. Vigilant neurovascular monitoring and proper splint application are essential to prevent pressure necrosis, compartment syndrome, and other complications. Integrating these evidence-based practices into a standardized approach to emergency ankle fracture management has the potential to reduce complications, improve functional outcomes, and limit unnecessary healthcare expenditures.
